# Subscapularis tendon tear classification and diagnosis: A systemic review and meta-analysis

**DOI:** 10.3389/fsurg.2023.916694

**Published:** 2023-03-15

**Authors:** Hossein Saremi, Mohamadali seifrabiei

**Affiliations:** ^1^ Department of Orthopedics, Faculty of Medicine, Hamadan University of Medical Sciences, Hamadan, Iran; ^2^ Social Medicine Department, Community Medicine Specialist, Faculty of Medicine, Hamadan University of Medical Sciences, Hamadan, Iran

**Keywords:** subscapularis tendon tear, MRI, MR arthrography, ultrasonography, clinical examination, CT arthrography

## Abstract

**Background:**

In the current study, we performed a systematic review and meta-analysis regarding the comparison of accuracy, sensitivity, and specificity of the techniques in diagnosing SSC tendon tears. Also, we performed a systematic review of the classification of SSC tendon tears.

**Methods:**

English language, peer-reviewed journal publications from the first date available to March 2022 were extracted by searching PubMed and Web of Science databases. A forest plot was used to graphically show the results of pooled sensitivity, specificity, and accuracy of different diagnostic modalities.

**Results:**

There were six studies on using MRI to diagnose subscapularis tendon tears, five studies on MRI, four studies on clinical examination, one on ultrasonography, and one on CT arthrography. Pooled sensitivity values for MRI, MRA, clinical examination, ultrasonography, and CT arthrography were 0.71 (CI: 0.54; 0.87), 0.83 (0.77; 0.88), 0.49 (0.31; 0.67), 0.39 (0.29; 0.51), and 0.90 (0.72–0.97), respectively. The pooled specificity values for MRI, MRA, clinical examination, ultrasonography, and CT arthrography were 0.93 (CI: 0.89; 0.96), 0.86 (0.75; 0.93), 0.89 (0.73; 0.96), 0.93 (0.88; 0.96), and 0.90 (0.69; 0.98), respectively. The pooled diagnostic accuracy values for MRI, MRA, clinical examination, ultrasonography, and CT arthrography were 0.84 (CI: 0.80; 0.88), 0.85 (0.77; 0.90), 0.76 (0.66; 0.84), 0.76 (0.70; 0.81), and 0.90 (0.78; 0.96), respectively.

**Conclusion:**

According to our systematic review and meta-analysis, MR arthrography was the most accurate in diagnosing subscapularis tears. MR arthrography was the most sensitive, and MRI and ultrasonography were the most specific in detecting subscapularis tears.

## Background

The rotator cuff muscle group is formed by the subscapularis (SSC) muscle, supraspinatus muscle, infraspinatus muscle, and teres minor muscle at the posterior scapular region. The SSC muscle originates from the subscapularis fossa of the scapula, inserts into the lesser tubercle of the humerus, and is innervated by the superior and inferior subscapular nerves. The SSC muscle is the largest component of the aforementioned rotator cuff; it is responsible for the elevation and internal rotation of the shoulder and has a crucial role in stabilizing the glenohumeral joint (1). As the subscapularis tendon tear is a prevalent painful condition followed by a significant loss of function, timely diagnosis and management of this condition are of crucial importance (2). Until now, several classifications have been proposed by Fox et al., Lyons, Lafosse et al., and Touissant et al. (3–6). However, there is no consensus regarding the classification of this condition, which may hinder clinical evaluation, diagnosis, and management.

The most sensitive and specific clinical examinations to assess an SCC tendon tear are the lift-off test, the belly-press test, and the bear-hug test. Increased external rotation compared to normal shoulder rotation and weakness in internal rotation also help in the diagnosis (7). Although these tests are essential for the diagnosis of SSC tendon tears, various imaging modalities such as magnetic resonance imaging (MRI), computed tomography (CT) scanning, magnetic resonance arthrography (MRA), and ultrasonography (US) may also be indicated (8). Since the SCC plays a crucial role in shoulder function, evaluating diagnostic modalities is of great significance. Misdiagnosed SSC tendon tears may result in unbalanced force, persistent shoulder pain, and weakness even after cuff repair (9). The gold standard diagnostic test for SSC tendon tears is arthroscopy, which helps the physician precisely evaluate the humeral and glenoid aspects of the joint space (10). As arthroscopy is an invasive, technically demanding, and expensive technique, using the aforementioned clinical assessments and imaging modalities can replace the necessity of performing an arthroscopy to diagnose SCC tendon tears. While several studies have provided evidence-based guidelines for the examination, diagnosis, and management of SSC tendon tears, no previous systematic review and meta-analysis studies have evaluated the diagnostic accuracy of MRI, MRA, CT scanning, US, and clinical assessments in the diagnosis of SSC tendon tears. Also, as mentioned earlier in the manuscript, a consensus regarding the classification of SSC tendon tears is still lacking.

In the current study, we performed a systematic review and meta-analysis regarding the comparison of accuracy, sensitivity, and specificity of these techniques in diagnosing SSC tendon tears. Also, we performed a systematic review of the classification of SSC tendon tears.

## Methods

We conducted a systematic review of the pieces of evidence for the diagnosis of subscapular tears with different diagnostic tools and studies on classification methods for subscapular tears. English language, peer-reviewed journal publications from the first date available to March 2022 were extracted by searching PubMed and Web of Science databases. The combination of the following search terms was used: subscapularis tear, diagnosis, and classification. After removing duplicate studies, the retrieved records were screened for title and abstract. The full text of eligible studies, selected from the previous step, was screened and reviewed. The data of interest were the sensitivity, specificity, and accuracy of different diagnostic tools in subscapularis tendon tears.

The eligible studies were observational studies (cross-sectional and cohort studies) on the diagnostic accuracy of imaging modalities and clinical assessments in subscapularis tendon tears. We excluded the studies that have one or more of the following criteria: (1) studies that were on rotator cuff tendons other than the subscapularis tendon (e.g., supraspinatus); (2) systematic reviews, meta-analysis studies, reviews, case reports, case series, and gray literature; (3) studies with no control group; (4) studies where the number of patients with subscapularis tendon tears was not specified; and (5) full text in any language other than English. For meta-analysis, we also excluded studies as reference tests other than arthroscopy (e.g., MRI or MR arthrography) because arthroscopy is the gold standard for diagnosing subscapularis tendon tears.

For data analysis, we used a meta package in R statistical software (version 4.1.1). The sensitivity, specificity, and accuracy of different diagnostic tests were calculated with a 95% confidence interval (CI). Sensitivity was considered as true-positive cases divided by total patients with a subscapularis tendon tear; specificity was considered as true-negative cases divided by total cases with an intact subscapularis tendon (confirmed by arthroscopy). Accuracy was considered as true-positive and true-negative cases divided by total subjects in the study. The random-effects model was used for calculating pooled sensitivity, specificity, and accuracy. In this study, subgroup analysis was used to report pooled sensitivity, specificity, and accuracy for different diagnostic tools. The forest plot was used to graphically represent the results of calculated pooled sensitivity, specificity, and accuracy for different subgroups. The *I*^2^ statistic was used to evaluate the heterogeneity in the included studies for each subgroup.

## Results

**Figure 1:**
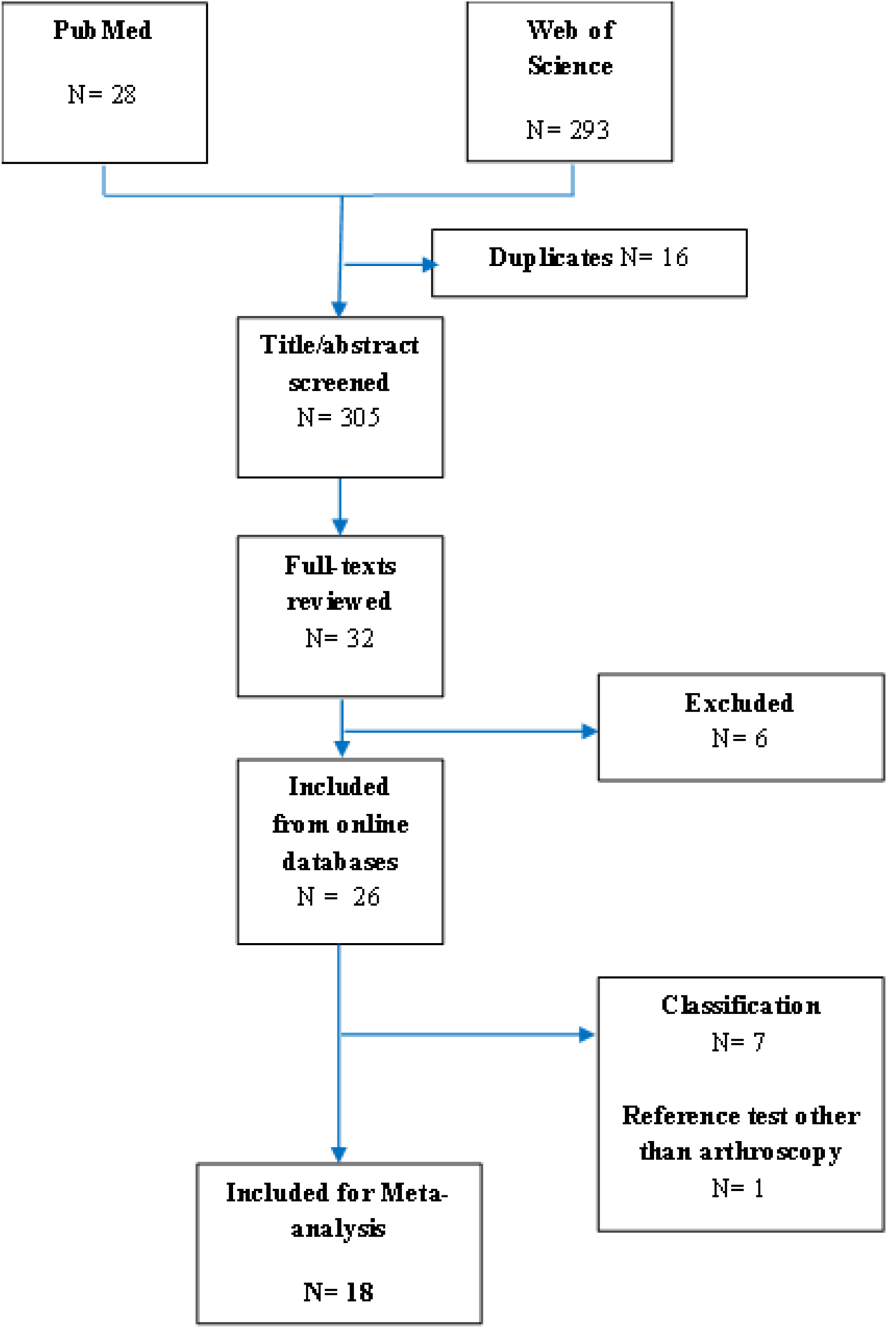
Flow diagram of the selection process.

The flow diagram of selected studies is shown in Figure 1. After reming duplicate records, 305 studies retrieved from online databases were screened for title andabstract. Thirty-two studies accomplished the inclusion criteria for full-text review. Six studies were excluded, leading to a final inclusion of 26 studies. The characteristics of the selected studies are presented in Table 1. Among them, seven studies were on the classification of the subscapularis tendon tear and 19 studies were on the diagnosis of subscapularis tendon tear. One study was excluded from meta-analysis because the reference test was MRI and not arthroscopy. Eighteen studies were included in our meta-analysis, and of 2,593 total subjects, 892 had subscapularis tendon tears.

**Table 1:** Characteristics of included studies.

Author	Year	Study design	Diagnosis modality	Mean age	Number of patients	STT	TP	TN	FP	FN	Reference test
MR arthrography											
Khil^a^ (11)	2020	Retrospective	MRA	63.1	180	31	27	146	3	4	Arthroscopy
Khil^b^ (11)	2020	Retrospective	MRA	62.8	241	92	68	121	28	24	Arthroscopy
Jung^c^ (12)	2017	Retrospective	MRA	57	84	30	24	39	15	6	Arthroscopy
Jung^d^ (12)	2017	Retrospective	MRA	56	83	40	30	38	5	10	Arthroscopy
Oh (13)	2009	Prospective	MRA	55	36	21	17	12	3	4	Arthroscopy
Choo^e^ (14)	2012	Retrospective	MRA	57.9	49	21	19	20	8	2	Arthroscopy
Choo^f^ (14)	2012	Retrospective	MRA	57.9	49	21	19	19	9	2	Arthroscopy
Lee (15)	2014	Retrospective	MRA	Median: 54	112	67	60	43	2	7	Arthroscopy
MRI											
Saremi (16)	2019	Retrospective	MRI	57.67	85	41	16	44	0	25	Arthroscopy
Atinga (17)	2021	Retrospective	MRI	56	55	19	12	34	2	7	Arthroscopy
Lee (18)	2019	Retrospective	MRI	Median: 57	112	67	51	43	2	16	Arthroscopy
Gyftopoulos (19)	2013	Retrospective	MRI	48	244	25	20	199	20	5	Arthroscopy
Malavolta (20)	2016	Retrospective	MRI	NA	93	50	39	37	6	11	Arthroscopy
Matsushita^g^ (21)	2022	Retrospective	MRI	NA	196	53	24	138	5	28	Arthroscopy
Matsushita^h^ (21)	2022	Retrospective	MRI	NA	196	53	49	126	17	4	Arthroscopy
Clinical examination											
Bartsch (22)	2010	Prospective	Clinical examination	58	50	15	6	23	6	9	Arthroscopy
Somerville (23)	2014	Prospective	Clinical examination	46	139	19	4	105	4	15	Arthroscopy with MRA
Lin (24)	2015	Prospective	Clinical examination	51	235	78	47	85	39	31	Arthroscopy
Takeda (25)	2016	Prospective	Clinical examination	65	130	46	30	69	4	16	Arthroscopy
Yoon (26)	2013	Retrospective	Clinical examination	57	312	133	16	179	0	117	MRI
Ultrasonography											
Narasimhan (27)	2016	Retrospective	Ultrasonography	NA	236	74	29	151	11	45	Arthroscopy
CT arthrography											
Asmar (28)	2020	Prospective	CT arthrography	54.1	50	29	26	19	2	3	Arthroscopy

MRI, magnetic resonance imaging; MRA, magnetic resonance arthrography; STT, subscapularis tendon tear; TP, true positive; TN, true negative; FP, false positive; FN, false negative.

^a^ Full-thickness tear.

^b^ Partial thickness tear.

^c^ Anterior approach.

^d^ Posterior approach.

^e^ Two dimensional.

^f^ Three dimensional.

^g^ cMRI.

^h^ rMRI.

### Accuracy of different types of methods in the diagnosis of subscapularis tendon tears

**Figure 2:**
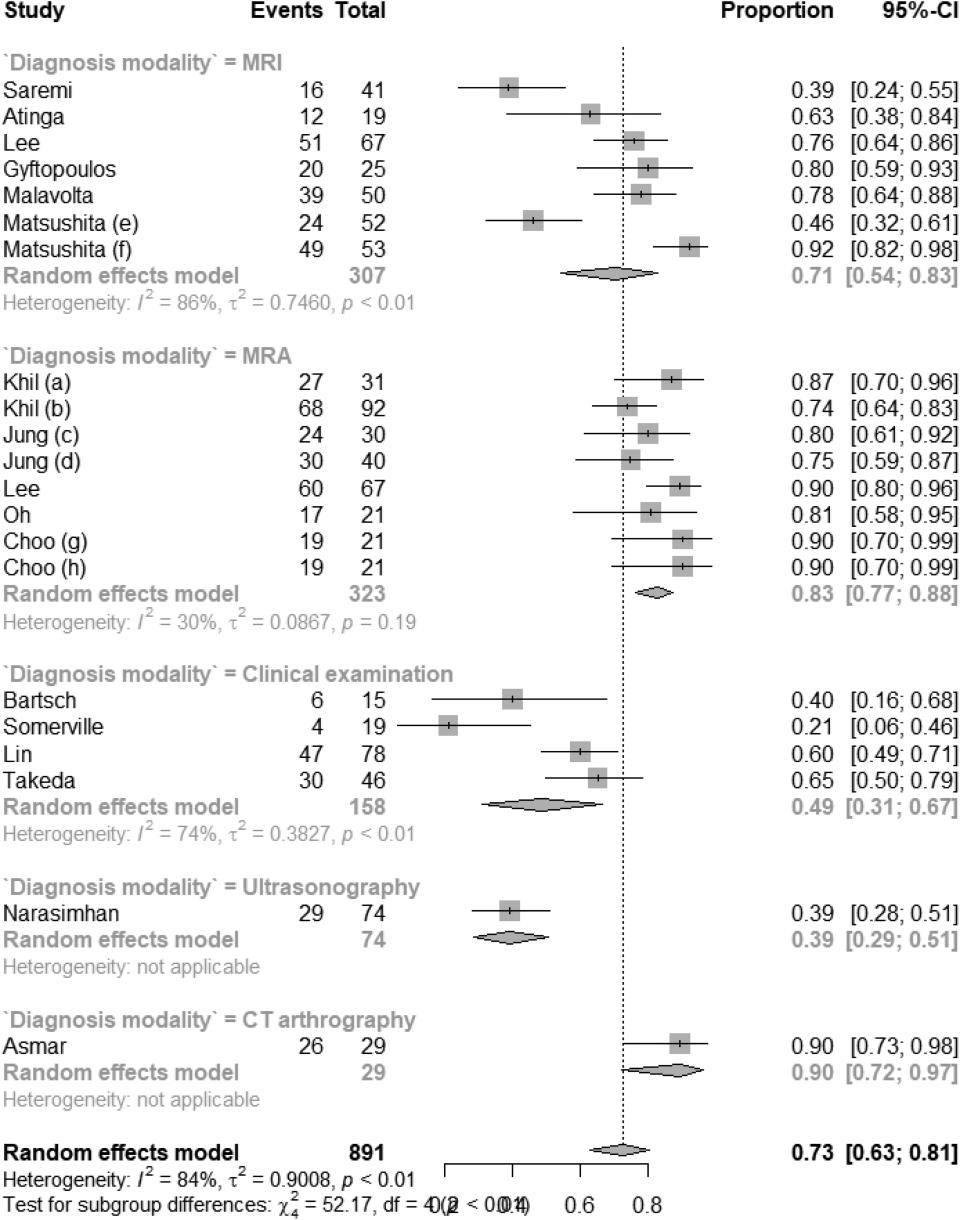
Forest plot of sensitivity of different diagnostic modalities.

**Figure 3:**
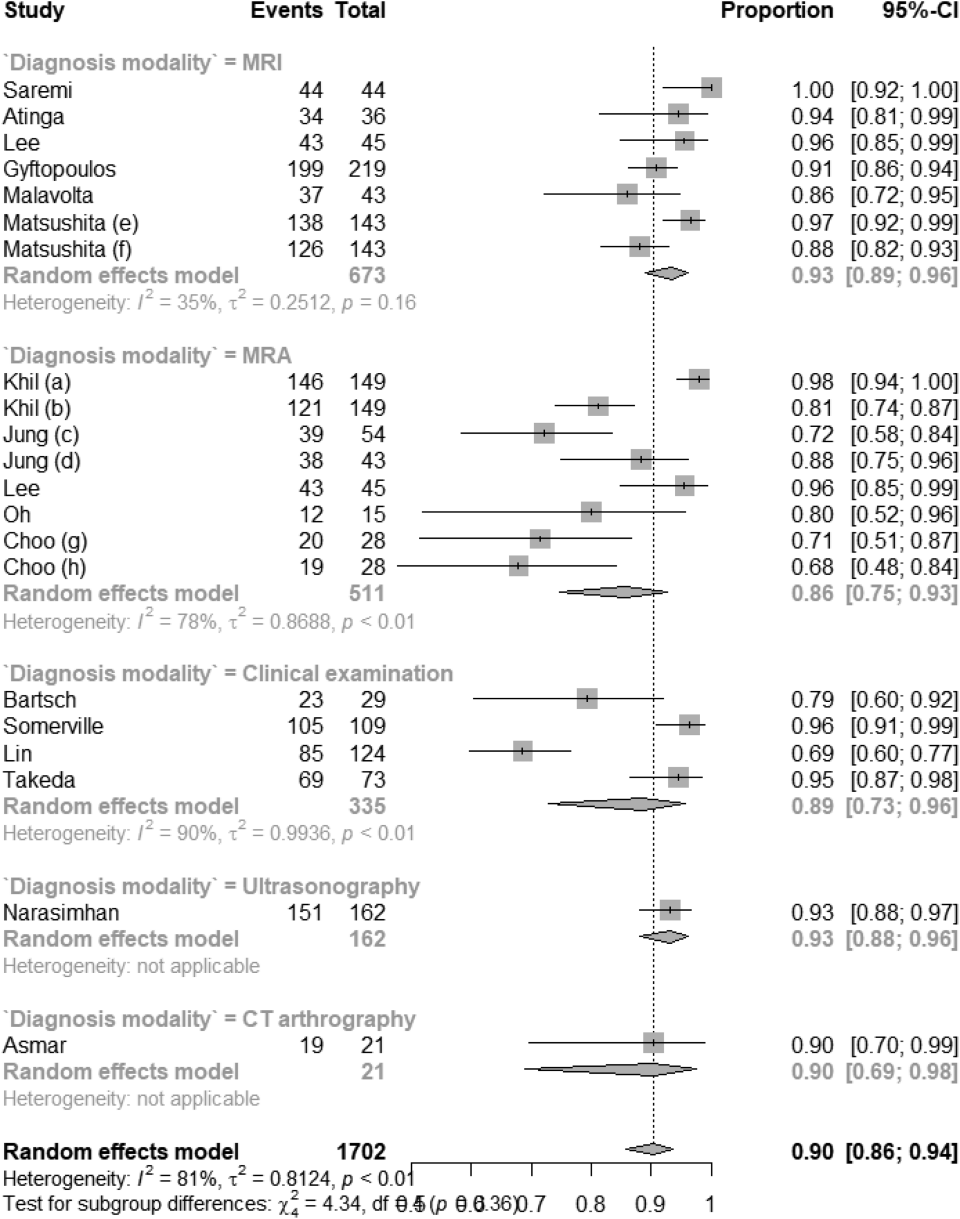
Forest plot of specificity of different diagnostic modalities.

**Figure 4:**
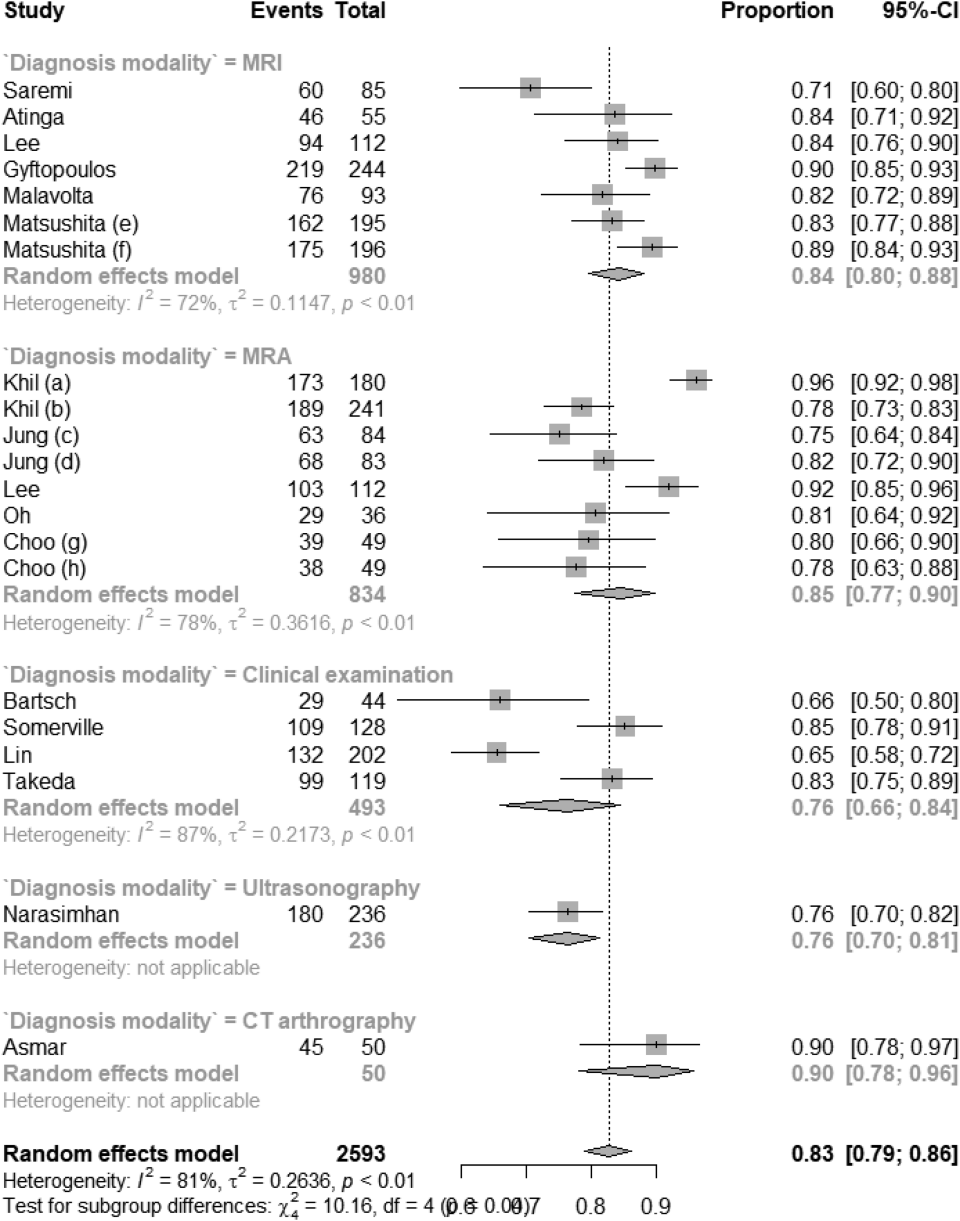
Forest plot of diagnostic accuracy of different diagnostic modalities.

There were six studies on using MRI to diagnose subscapularis tendon tears, five studies on MRI, four studies on clinical examination, one on ultrasonography, and one on CT arthrography. As shown in Figure 2, the pooled sensitivity values for MRI, MRA, clinical examination, ultrasonography, and CT arthrography were 0.71 (CI: 0.54; 0.87), 0.83 (0.77; 0.88), 0.49 (0.31; 0.67), 0.39 (0.29; 0.51), and 0.90 (0.72–0.97), respectively. The *I*^2^ statistic for each subgroup is shown in Figure 2. The pooled specificity values for MRI, MRA, clinical examination, ultrasonography, and CT arthrography were 0.93 (CI: 0.89; 0.96), 0.86 (0.75; 0.93), 0.89 (0.73; 0.96), 0.93 (0.88; 0.96), and 0.90 (0.69; 0.98), respectively. The *I*^2^ statistic for each subgroup is shown in Figure 3. The pooled diagnostic accuracy values for MRI, MRA, clinical examination, and CT arthrography were 0.84 (CI: 0.80; 0.88), 0.85 (0.77; 0.90), 0.76 (0.66; 0.84), 0.76 (0.70; 0.81), and 0.90 (0.78; 0.96) respectively. The *I*^2^ statistic for each subgroup is shown in Figure 4.

### Classification of subscapularis tendon tears

Seven studies were found on the classification of subscapularis tendon tears (3–6, 29, 30). The different classification types are listed in Table 2.

**Table 2:** Different classification systems.

Type	Description
Lafosse’s classification	
I	Partial lesion of superior one-third
II	Complete lesion of superior one-third
III	Complete lesion of superior two-thirds
IV	Complete lesion with centered head and fatty degeneration < stage3
V	Complete lesion with eccentric head and fatty degeneration > stage3
Yoo and Rhee’s subscapularis tendon tear classification	
I	Fraying or longitudinal split of the subscapularis leading-edge tendon
IIA	<50% subscapularis tendon detachment in the first facet (concealed lesion included)
IIB	>50% detachment in the first facet without complete disruption of the lateral band (concealed lesion included)
III	Entire first facet with complete disruption of the lateral band (full-thickness tear of upper one-third of the subscapularis superior–inferior length)
IV	Up to the second facet tear: the first and second facets are exposed with much more medial retraction of the tendon, which is approximately a two-thirds tear of the entire subscapularis superior–inferior length (the entire tendinous portion)
V	Complete subscapularis tendon tear involving the muscular portion
Fox and Romeo’s classification	
I	Partial-thickness tear
II	Complete tear of upper 25%
III	Complete tear of upper 50%
IV	Complete rupture
Martetschläger’s classification (for partial tears)	
I	Split lesion
II	Tear smaller than 10 mm
III	Tear between 10 and 15 mm
IV	Tear larger than 15 mm
Lyons's classification	
I	Partial thickness, partial length
II	Full thickness, partial length
III	Full thickness, full length without retraction
IV	Full thickness, full length with retraction
Toussaint’s classification	
I	Partial tendon tear with intact bicipital sling
II	Partial tendon tear with partial bicipital sling injury with intact SGHL
III	Complete tendon tear with complete bicipital sling injury, minimal tendon retraction
IV	Complete tendon tear with complete bicipital sling injury, with retraction
Dierckman’s classification	
I	Distinct, isolated nodule found on the leading edge of the subscapularis tendon with minimal degeneration
II	Longitudinal split tear of the upper ½ of the tendon without significant degeneration
III	Longitudinal tear of the upper ½ of the tendon with significant degeneration and fibrillation

## Discussion

To our knowledge, this is the first systematic review and meta-analysis comparing the accuracy of different methods in diagnosing subscapularis tears. Our systematic review and meta-analysis showed that MR arthrography and CT arthrography were the most accuracte in diagnosing subscapularis tears, with accuracy values of 85% and 90%, respectively. However, there was only one study on the diagnostic accuracy of CT arthrography, and one is not enough to conclude. MRA and CT arthrography were the most sensitive, with a sensitivity of 83% for MR arthrography and 90% for CT arthrography. Again, the results could not be reliable due to the low sample size in CT arthrography. MRI and ultrasonography were the most specific in detecting subscapularis tears, with a specificity of 93% for both of them. However, ultrasonography has much lower sensitivity, resulting in lower accuracy than MRI.

Clinical assessment and imaging studies are the two main methods for diagnosing subscapularis tendon tears (9). Generally, our meta-analysis showed that imaging studies are more accurate, sensitive, and specific than clinical assessment in the diagnosis of subscapularis tendon tears. However, clinical assessments could have high specificity in detecting such disorders. The patients may report weakness in internal rotation but is nonspecific of a subscapularis tendon tear. In our study, the clinical test used for the diagnosis of subscapularis tendon tear was the lift-off test; a previous meta-analysis of the lift-off test by Lädermann et al. showed that it has the highest accuracy among all clinical tests (31). However, clinical tests are subjective. The subscapularis tendon strength can be assessed by a dynamometer, which provides force comparison with the contralateral shoulder and objective values (9).

Ultrasonography is among other imaging techniques for the diagnosis of subscapularis tendon disorders . Although there are numerous studies on the accuracy of ultrasonography for diagnosing rotator cuff tendon tears particularly supraspinatus tendon tears, there are limited studies on the accuracy of ultrasonography in diagnosing subscapularis tendon tears (32). A meta-analysis conducted by Farooqi et al. on the diagnostic accuracy of ultrasonography for rotator cuff tears showed that ultrasonography has a more diagnostic accuracy for bicep tendon tears (93%) and supraspinatus tendon tears (83%) compared to subscapularis tendon tears (76%). However, ultrasonography is highly specific in detecting subscapularis tendon tears (93%) compared to other imaging modalities. Thus, a positive result could be considered for subsequent definitive diagnosis and management procedures such as arthroscopy, but a negative result needs more diagnostic tests to approve. Further studies on assessing the diagnostic accuracy of ultrasonography for subscapularis tendon tears are required to make more definitive conclusions.

MRI and MR arthrography are more reliable compared to ultrasonography or clinical assessment for the diagnosis of subscapularis tendon tears (33). A meta-analysis conducted by Malavolta et al. on the efficacy of MRI and MR arthrography in the diagnosis of subscapularis tendon tears showed that the pooled sensitivity and specificity of MRI and MR arthrography in the diagnosis of the subscapularis tendon tears were 68% and 90%, respectively. However, this study did not conduct a separate meta-analysis for MRI and MR arthrography (33). Our study is in line with this meta-analysis as the sensitivity of MRI and MR arthrography is lower than their specificity, which means that MRI and MR arthrography have lower false-positive cases than false-negative cases. Previous studies show that the diagnostic value of MRI is higher in a complete tear of the subscapularis tendon (Type 4 by the Lafosse classification) (16) and the tear of the other rotator cuff tendons (34). The relatively lower sensitivity of MRI and MR arthrography for subscapularis tendon tears than that for other rotator cuff tendons could be explained by its three-dimensional footprint topography of the humeral head, which is explained by Yoo et al. (30), or by some noninsertional types of subscapularis tears (35). $$$$The accuracy also increases with the higher expertise of the reviewer (36). On the other hand, the diagnostic accuracy of MRI and MR arthrography is not affected by the time elapsed from injury to perform the imaging study (16).

Considering the accuracy of each diagnostic tool is important to obtain good clinical and functional outcomes in the treatment of subscapularis tendon tears (37, 38), although treatment of elderly patients is often limited surgically due to the bad quality of the tissue. Hence, a shoulder replacement could be indicated (39).

**Figure 5:**
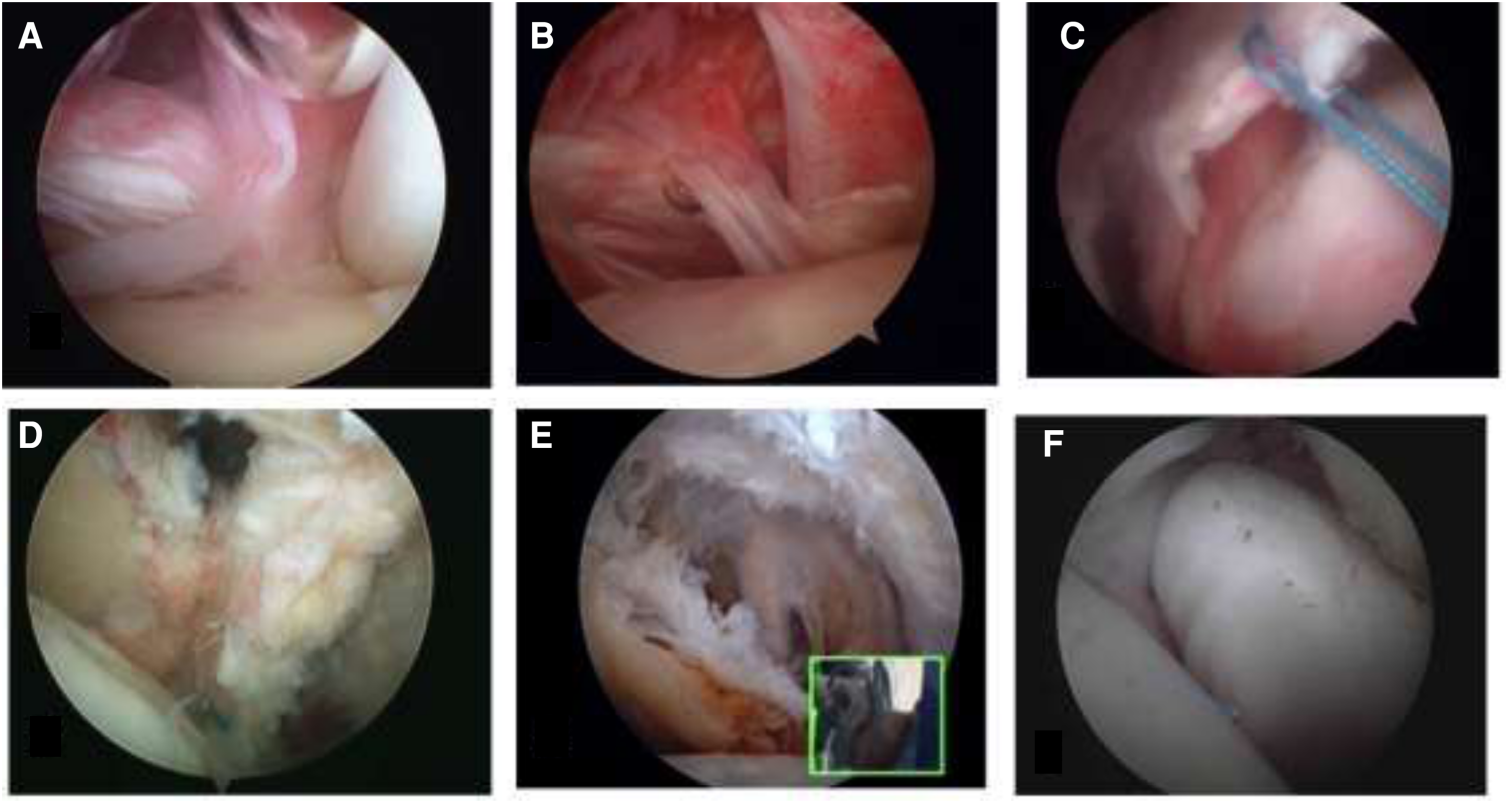
Arthroscopic view of the Lafosse classification of subscapularis tendon tears (**A**–**D**) (4), airbag sign, an arthroscopic finding of interstitial tears of the subscapularis tendon (35).

Classifications of Lafosse (Figures 5A-E), Fox, Lyons, Martetschlager, and Toussaint are based on the insertion site lesions and according to anatomic data and arthroscopic lesion-related findings (3–6, 29). Yoo et al. described a classification based on a three-dimensional anatomic footprint (30). Dierckman’s classification was based on noninsertional tendinopathy of the subscapularis (40). None of the classifications included interstitial tears of the subscapularis tendon described by Saremi et al. (Figure 5F) (35).

The main strengths of this study are as follows: (1) this is the first systematic review and meta-analysis comparing the accuracy of different methods in diagnosing subscapularis tears; (2) this study compared not only the different imaging modalities but also the accuracy of imaging modalities with clinical examinations; and (3) we conducted a meta-analysis of the sensitivity and specificity in addition to the accuracy of different diagnostic modalities.

The limitation of this study is that we did not compare the specific clinical tests, and we also did not compare complete and partial tears of the subscapularis tendon tear. However, adding another level of subgroups could have affected the possibility of conducting a meta-analysis.

## Conclusion

According to our systematic review and meta-analysis, MR arthrography was the most accurate in diagnosing subscapularis tears, MR arthrography was the most sensitive, and MRI and ultrasonography were the most specific in detecting subscapularis tears. Further studies on assessing the diagnostic accuracy of ultrasonography and CT arthrography for subscapularis tendon tears are required to make more definitive conclusions.

## Data Availability

The original contributions presented in the study are included in the article/Supplementary Material, further inquiries can be directed to the corresponding author.
